# Assessment of diagnosis of inflammatory breast cancer cases at two cancer centers in Egypt and Tunisia

**DOI:** 10.1002/cam4.48

**Published:** 2013-01-24

**Authors:** Catherine Schairer, Amr S Soliman, Sherif Omar, Hussein Khaled, Saad Eissa, Farhat Ben Ayed, Samir Khalafallah, Wided Ben Ayoub, Elizabeth D Kantor, Sofia Merajver, Sandra M Swain, Mitchell Gail, Linda Morris Brown

**Affiliations:** 1 Division of Cancer Epidemiology and Genetics, National Cancer Institute6120 Executive Blvd, Rockville, Maryland, 208522010;7234; 2 Department of Epidemiology, College of Public Health, University of Nebraska Medical CenterOmaha, Nebraska, 68198-4355; 3 Department of Surgery, National Cancer Institute, Cairo UniversityFom El Khalig Sq, Cairo, 11796, Egypt; 4 National Cancer Institute, Cairo UniversityFom El Khalig Sq, Cairo, 11796, Egypt; 5 Pathology Department, National Cancer Institute, Cairo UniversityFom El Khalig Sq, Cairo, 11796, Egypt; 6 Institut Salah AzaizBoulevard du 9 Avril, Bab-Sâadoun, Tunis, 1006, Tunisia; 7 School of Public Health, University of Michigan1415 Washington Heights, Ann Arbor, Michigan, 48109-2029; 8 Department of Internal Medicine, University of Michigan Medical SchoolAnn Arbor, Michigan, 48109-2029; 9 Medical Oncology Branch, National Naval Medical Center8901 Wisconsin Avenue, Bldg 8, Room 5101, Bethesda, Maryland, 20889-5105

**Keywords:** Edema, Egypt, erythema, inflammatory breast cancer, peau d'orange, Tunisia

## Abstract

The diagnosis of inflammatory breast cancer (IBC) is largely clinical and therefore inherently somewhat subjective. The objective of this study was to evaluate the diagnosis of IBC at two centers in North Africa where a higher proportion of breast cancer is diagnosed as IBC than in the United States (U.S.). Physicians prospectively enrolled suspected IBC cases at the National Cancer Institute (NCI) – Cairo, Egypt, and the Institut Salah Azaiz (ISA), Tunisia, recorded extent and duration of signs/symptoms of IBC on standardized forms, and took digital photographs of the breast. After second-level review at study hospitals, photographs and clinical information for confirmed IBC cases were reviewed by two U.S. oncologists. We calculated percent agreement between study hospital and U.S. oncologist diagnoses. Among cases confirmed by at least one U.S. oncologist, we calculated median extent and duration of signs and Spearman correlations. At least one U.S. oncologist confirmed the IBC diagnosis for 69% (39/50) of cases with photographs at the NCI-Cairo and 88% (21/24) of cases at the ISA. All confirmed cases had at least one sign of IBC (erythema, edema, peau d'orange) that covered at least one-third of the breast. The median duration of signs ranged from 1 to 3 months; extent and duration of signs were not statistically significantly correlated. From the above-mentioned outcomes, it can be concluded that the diagnosis of a substantial proportion of IBC cases is unambiguous, but a subset is difficult to distinguish from other types of locally advanced breast cancer. Among confirmed cases, the extent of signs was not related to delay in diagnosis.

## Introduction

Inflammatory breast cancer (IBC) is a rare, aggressive form of breast cancer characterized by the rapid clinical appearance of erythema (redness), edema, and peau d'orange of the breast thought to result from the presence of tumor emboli in the breast dermal lymphatics. The diagnosis of IBC is primarily clinical; however, a tissue diagnosis is still necessary to establish invasive breast cancer. The extent and duration of clinical signs required for IBC has not been well standardized 1; the seventh edition of the American Joint Committee on Cancer (AJCC) suggests that diffuse erythema and edema (peau d'orange) should involve a third or more of the breast 2. Neglected locally advanced breast cancers presenting late in the course of disease are not to be considered IBC 2.

Inflammatory breast cancer has been reported to constitute a larger proportion of breast cancers in North Africa than in the United States (U.S.) 3,4, although the proportion of IBC in some parts of North Africa appears to be declining 5. Because the diagnosis of IBC is largely clinical and therefore inherently somewhat subjective, it may be difficult to distinguish IBC from other types of locally advanced breast cancers, particularly in areas such as North Africa where the majority of breast cancers are diagnosed at an advanced stage 6–8. Therefore, we undertook a pilot study to prospectively assess the diagnostic criteria for IBC at two major cancer centers in North Africa, the National Cancer Institute (NCI) – Cairo, Egypt, and the Institut Salah Azaiz in Tunisia, with further review via photographs and clinical information by two oncologists in the U.S. We present detailed information on signs/symptoms associated with IBC and their duration. To our knowledge, this is the first such prospective and systematic assessment of the diagnosis of IBC using photographs and external review. Previous assessments of the diagnosis of IBC in North Africa have relied on retrospectively reviewed information provided in medical records 3,5.

## Materials and Methods

Female breast cancer cases exhibiting erythema, edema, or peau d'orange and determined to be IBC by examining physicians in two of three oncology clinics in the Department of Medical Oncology (NCI-Cairo) between February 12 and September 28, 2005, and the Department of Surgery at the Institut Salah Azaiz, Tunis, Tunisia (ISA), from January 31 to October 25, 2005, were enrolled in a study to systematically record their clinical characteristics before treatment. All cases were diagnosed pathologically as breast cancer. Standardized forms were used by the clinicians to record the presence, extent, and duration of erythema, edema, peau d'orange, ulceration, tumor size, and other clinical signs and symptoms at the time of patient examination. Patients were also asked to consent to digital photographs of the breast. Cases at NCI-Cairo were further reviewed by a senior clinician to verify IBC status, whereas cases at the ISA were reviewed and verified as IBC during weekly cancer committee meetings.

To further assess the IBC diagnosis, two physicians in the U.S. (SMS and SM) with expertise in IBC examined digital photographs of the breast in conjunction with clinical information. The U.S. physicians were board certified in oncology and experienced in evaluating patients with all forms of breast cancer and IBC in particular. They evaluated the photographs for characteristics of IBC, such as presence of erythema, edema, and peau d'orange, and used their clinical judgment to distinguish these signs from those associated with other types of locally advanced breast cancer 9. IRB approval for the study was obtained from the NCI-U.S. and participating institutions.

Using SAS version 9.2 (SAS Institute, Inc., Cary, NC), we calculated Spearman rank correlation coefficients (*r*) and derived *P*-values from the *t*-statistic 
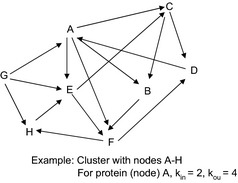
, which were compared to critical values of the *t*-distribution with *n* − 2 degrees of freedom 10. A two-sided *P*-value ≤0.05 was considered statistically significant.

## Results

### Concordance between study hospital diagnosis and U.S. review

**Figure 1:**
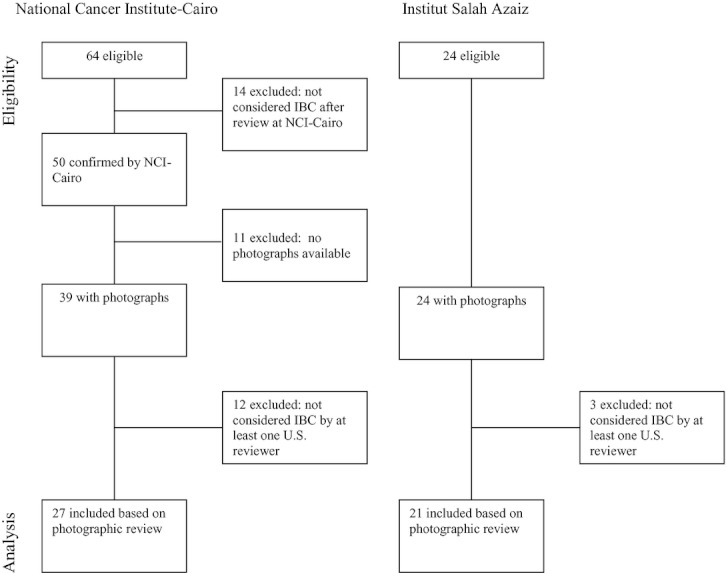
Criteria for study inclusion.

The accrual of cases in the study is shown in Figure 1. A total of 64 breast cancers seen at the NCI-Cairo during the study period were initially classified as IBC; 50 (78%) were confirmed as IBC upon second-level review at the NCI-Cairo. Digital photographs were obtained for 39 of these 50 cases (78%), of which 27 (69%) were considered to be IBC by at least one of two physician reviewers in the U.S. and 17 (44%) were considered IBC by both U.S. reviewers. A total of 24 breast cancer cases were identified as IBC by physicians at the Institut Salah Azaiz in Tunisia during the study period. All 24 were confirmed as IBC upon second-level review at the ISA. Photographs were available for all 24 cases; 21 (88%) were considered to be IBC by at least one of two reviewers in the U.S., and 18 of the 24 (75%) were considered IBC by both U.S. reviewers.

**Figure 2:**
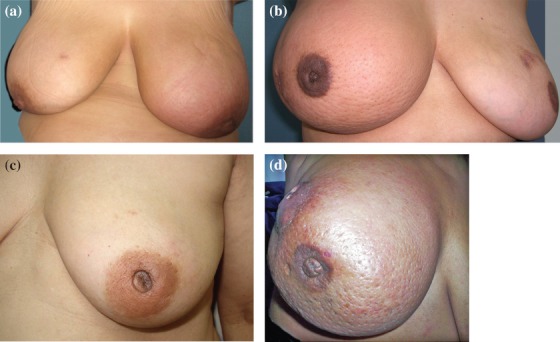
(a) All raters agreed that this is an inflammatory breast cancer (IBC) case; erythema (50%), edema (60%), and peau d'orange (80%) of the left breast, no tumor mass, duration of signs is 2 weeks. (b) All raters agreed this is an IBC case; erythema (60%), edema (80%), and peau d'orange (80%) of the right breast, no tumor mass, duration of signs is 1 month; (c). Study hospital considered this an IBC case; one U.S. rater agreed, the other did not; erythema (10%), edema (40%), peau d'orange (40%) of the left breast, duration of signs is 12 months. (d) Study hospital considered this an IBC case; both U.S. raters did not; erythema (60%), edema (100%), peau d'orange (100%) of the right breast, duration of signs 72 months. Percentages of the breast covered by redness, edema, and peau d'orange are based on the clinical examination form completed by the study hospital physicians.

Photographs of selected cases are shown in Figure 2, including two cases that all raters, including the two U.S. oncologists, considered to be IBC (Fig. 2a and b), one case considered to be IBC by the study hospital but considered to be ambiguous by the U.S. oncologists due to the absence of extensive redness (Fig. 2c), and one case considered to be IBC by the study hospital, but considered to be a neglected non-IBC locally advanced breast cancer by both U.S. raters (Fig. 2d).

### Characteristics of cases designated as IBC by study hospital and at least one U.S. reviewer

Subsequent analyses are limited to the 27 cases at the NCI-Cairo and 21 cases at the ISA considered to be IBC after second level of review at the study institutions in North Africa as well as by at least one U.S. reviewer. The mean age at diagnosis of IBC cases was 50.3 years at the NCI-Cairo and 47.8 years at the ISA. Other characteristics of the IBC cases are shown in Table 1. The vast majority of cases at both study sites had erythema, edema, and peau d'orange of the breast, swollen axillary lymph nodes, breast warmth, ridges/thickening of the breast, and flattened/inverted nipples. A higher percentage of cases at the NCI-Cairo had an underlying tumor mass, pain/tenderness, itching/pruritis, or change in the color/texture of the areola. Only a few cases at either center had ulceration of the breast or nipple discharge. The median percent of the breast presenting with erythema, edema, and peau d'orange was 60% or greater at both study hospitals. All cases had at least one sign of IBC (erythema, edema, peau d'orange) that covered approximately one-third or more of the breast, consistent with the definition of IBC in the seventh edition of the AJCC (Table 2) 2.

**Table I:** Characteristics of inflammatory breast cancer cases

	National Cancer Institute – Cairo *n* (%)	Institut Salah Azaiz *n* (%)
Total cases	27 (100)	21 (100)
Menopausal status		
Premenopausal	12 (44)	13 (65)^1^
Perimenopausal	1 (4)	
Postmenopausal	14 (52)	7 (35)
Erythema	26 (96)	19 (90)
Edema	26 (96)	20 (95)
Peau d'orange	24 (89)^1^	18 (86)
Mass	22 (85)^1^	1 (5)^1^
Ulceration	4 (15)	1 (5)^1^
Swollen axillary nodes	26 (96)	18 (86)
Breast painful/tender	22 (81)	5 (24)
Breast warm	24 (89)	17 (81)
Breast itching/pruritis	19 (70)	12 (57)
Nipple discharge	5 (19)	0^1^
Ridges/thickening of breast	22 (81)	19 (90)
Nipple flattened/inverted	25 (93)	20 (95)
Change in color/texture of areola	21 (78)	12 (57)

1 Missing data for one subject; percentage of those with known data.

**Table 2:** Extent and duration of signs of erythema, edema, and peau d'orange

Sign	Extent of sign (% of breast) median (range)	Duration of sign (months) median (range)	Spearman correlation of extent and duration of signs	*P*-value for correlation statistic
National Cancer Institute – Cairo				
Erythema	70 (10–100)^1^	3.0 (0.5–13.0)^1^	0.05^1^	0.80
Edema	70 (20–100)^1^	2.5 (0.5–13.0)^1^	−0.15^1^	0.47
Peau d'orange	70 (30–100)^2^	3.0 (0.5–13.0)^2^	0.02^2^	0.92
Institut Salah Azaiz				
Erythema	60 (10–80)^3^	1.0 (0.5–12.0)^2^	−0.19^2^	0.45
Edema	70 (40–90)	1.5 (0.5–9.0)^3^	−0.45^2^	0.05
Peau d'orange	70 (50–90)^3^	1.5 (0.5–9.0)^2^	−0.25^2^	0.32

1 Missing data for one subject.

2 Missing data for three subjects.

3 Missing data for two subjects.

The median duration of these signs was 2.5–3 months in Egypt and 1–1.5 months in Tunisia. The physician-reported extent of signs (erythema, edema, and peau d'orange) and patient-reported duration of these signs were not significantly correlated (Table 2). Although not statistically significant, many of the correlations were negative, suggesting that those with more extensive redness, edema, and peau d'orange sought medical care earlier.

Positive correlations between the extent of erythema, edema, and peau d'orange were significant or approached statistical significance among NCI-Cairo cases; among ISA cases, only the extent of edema and peau d'orange were significantly positively correlated (Table 3).

**Table 3:** Spearman correlation coefficients (*P*-values) between extent of signs of erythema, edema, and peau d'orange

	Edema	Peau d'orange
National Cancer Institute – Cairo		
Erythema	0.56^1^ (0.004)*	0.39^2^ (0.07)*
Edema		0.52^3^ (0.01)*
Institut Salah Azaiz		
Erythema	0.25^1^ (0.30)*	0.28^1^ (0.27)*
Edema		0.83^1^ (<0.0001)*

1 Missing data for two subjects.

2 Missing data for four subjects.

3 Missing data for three subjects.

* *P*-value.

Among the 85% of NCI-Cairo cases with a mass, the median clinically determined tumor size was 8.0 cm (range 4–16 cm). Tumor size was not significantly correlated with extent of erythema (*r* = 0.29, *P* = 0.19), edema (*r* = 0.07, *P* = 0.75), or peau d'orange (*r* = 0.009, *P* = 0.97; data not shown in Table 3).

Results were not materially different when cases without photographs were included or cases with ulceration (which is sometimes associated with neglected breast cancer) were deleted. Further restriction of cases to those determined to be IBC by both U.S. reviewers resulted in a statistically significant negative correlation between duration and extent of edema for cases from the ISA (*r* = −0.73, *P* = 0.0014).

### Characteristics of cases designated as IBC by study hospital but not by at least one U.S. reviewer

The median age of the 12 cases considered to be IBC by the NCI-Cairo, but not by at least one U.S. clinician, was 61 years. The median percentages of the breast covered by redness, edema, and peau d'orange were 45, 60, and 55, respectively, and the median duration of signs was 12 months (range 0.5–72 months). All had a tumor mass. Median tumor size was 8.0 cm. Fifty-eight percent had ulcerations of the breast.

The median age of the three cases excluded at the ISA was 47 years. The median percentages of the breast covered by redness, edema, and peau d'orange were 100, 70, and 60, and the median duration of signs was 1–1.5 months (maximum 7 months). No cases had tumor masses. The skin of one excluded subject showed evidence of a chronic burn, and another subject had cancer en cuirasse.

## Discussion

In this pilot study, we did a systematic review of the diagnosis of IBC at two hospitals in North Africa (the NCI-Cairo, Egypt, and the ISA, Tunis, Tunisia), where a higher proportion of breast cancers have been reported to be IBC than in the U.S. We used digital photographs, detailed clinical information, and multiple levels of review.

Our study suggests that diagnostic criteria for IBC vary among physicians and institutions. We found generally good agreement among clinicians at the ISA in Tunisia and the collaborating physicians in the U.S. There was less consistency at the NCI-Cairo, with 22% of initially suspected IBC cases not confirmed upon further review at the study hospital; of those confirmed at the study hospital, 70% were considered to be IBC upon review in the U.S., with the remaining 30% considered to be other types of locally advanced breast cancers. At the NCI-Cairo, but not the ISA, the cases not confirmed by at least one U.S. oncologist tended to be older and to have a longer median duration of signs and more frequent breast ulcerations than cases that were confirmed by at least one U.S. physician.

The confirmed cases at both study hospitals had signs consistent with the SEER Collaborative Stage for TNM 7 codes 725, 730, and 750 for diagnosis of IBC (diagnosis of inflammatory carcinoma with a clinical description of erythema, edema, peau d'orange involving one-third or more, but less than or equal to half (50%) of the skin of the breast, involving more than 50% of the breast, and percent of breast not stated, respectively) 11. We found no evidence that confirmed cases with more extensive redness, edema, and peau d'orange had longer delays in seeking health care. In fact, there was some evidence that those with the most extensive signs sought medical attention earlier. Moreover, the median duration of signs before medical consultation among confirmed IBC cases in our study (1–3 months) was considerably lower than the mean delay of 11.6 months reported among 160 patients with breast cancer showing local or T4 disease in central Tunisia 12.

In a seminal description of clinical signs and symptoms of 89 IBC cases, Haagensen 13 noted redness in 57% of cases, breast enlargement in 48%, edema of the skin in 13%, a localized tumor in 57%, warmth of the skin in 8%, nipple retraction in 13%, nipple discharge in 8%, and pain in 29%. Studies in other populations have reported signs and symptoms that range in frequency between Haagensen's results and our results for the cases from NCI-Cairo 14,15–17. We are unaware of previous studies which have linked the extent and duration of signs in any study population.

The redness, edema, and peau d'orange characteristic of IBC are thought to derive from the presence of tumor emboli in the dermal lymphatic vessels 18, although the presence of these emboli is not part of the definition of IBC 2,18. In fact, a number of studies have shown that dermal lymphatic invasion is identified in less than 75% of IBC cases despite adequate sampling of skin 18. Twenty-six of the 27 NCI-Cairo IBC cases in this analysis that were confirmed as IBC by at least one of two reviewers in the U.S. were included in prior publications examining the presence of tumor emboli in the dermal lymphatics of Egyptian IBC cases 19,20. Forty-nine percent of these Egyptian IBC patients had such tumor emboli; the number of tumor emboli was not associated with the number or duration of symptoms or tumor size. Tumor tissues from the Tunisian cases included in this study were not available for assessment of the presence of tumor emboli.

Strengths of our study include the prospective collection of suspected IBC cases, the availability of photographs and systematically collected information on the extent and duration of signs and symptoms, and the two layers of review to confirm IBC diagnosis. We must note, however, that not all cases consented to be digitally photographed and that the external review was based on photographs which were sometimes difficult to interpret. It is also possible that the durations of signs were not accurately reported by some study participants. Although the same forms were used to collect information at both study sites, it is possible that the signs and symptoms were interpreted differently at the two sites. For instance, induration of the breast may have been considered a tumor mass in Egypt but not Tunisia, which may partially account for the notable difference in the percentage of cases with a tumor mass in Egypt and Tunisia. Finally, it remains unclear how neglected IBC can be distinguished for other types of neglected locally advanced breast cancers.

In summary, our results suggest that the clinical diagnosis of a substantial proportion of IBC cases is unambiguous, but in certain cases, it is difficult to distinguish IBC from other types of locally advanced breast cancer, particularly those that show extensive redness, edema, and peau d'orange of long duration (e.g. Fig. 2d), ulcerations, or those where it is ambiguous how much of the breast is affected by redness, edema, and peau d'orange (e.g. Fig. 2c). Therefore, we recommend that training to standardize IBC diagnosis, digital photographs, collection of detailed clinical information, and several layers of review be included in studies of IBC.
